# Global, regional, and national burden of motor neuron diseases 1990–2016: a systematic analysis for the Global Burden of Disease Study 2016

**DOI:** 10.1016/S1474-4422(18)30404-6

**Published:** 2018-12

**Authors:** GBD 2016 Motor Neuron Disease Collaborators, Giancarlo Logroscino, Marco Piccininni, Benoît Marin, Emma Nichols, Foad Abd-Allah, Ahmed Abdelalim, Fares Alahdab, Solomon Weldegebreal Asgedom, Ashish Awasthi, Yazan Chaiah, Ahmad Daryani, Huyen Phuc Do, Manisha Dubey, Alexis Elbaz, Sharareh Eskandarieh, Farzaneh Farhadi, Farshad Farzadfar, Seyed-Mohammad Fereshtehnejad, Eduarda Fernandes, Irina Filip, Kyle J. Foreman, Abadi Kahsu Gebre, Elena V. Gnedovskaya, Samer Hamidi, Simon I. Hay, Seyed Sina Naghibi Irvani, John S. Ji, Amir Kasaeian, Yun Jin Kim, Lorenzo Giovanni Mantovani, Tivani Phosa Mashamba-Thompson, Man Mohan Mehndiratta, Ali H. Mokdad, Gabriele Nagel, Trang Huyen Nguyen, Molly R Nixon, Andrew T Olagunju, Mayowa Ojo Owolabi, Michael A Piradov, Mostafa Qorbani, Amir Radfar, Robert C Reiner, Mohammad Ali Sahraian, Shahabeddin Sarvi, Mehdi Sharif, Omar Temsah, Bach Xuan Tran, Nu Thi Truong, Narayanaswamy Venketasubramanian, Andrea Sylvia Winkler, Ebrahim M Yimer, Valery L. Feigin, Theo Vos, Christopher J L Murray

## Abstract

**Background:**

Understanding how prevalence, incidence, and mortality of motor neuron diseases change over time and by location is crucial for understanding the causes of these disorders and for health-care planning. Our aim was to produce estimates of incidence, prevalence, and disability-adjusted life-years (DALYs) for motor neuron diseases for 195 countries and territories from 1990 to 2016 as part of the Global Burden of Diseases, Injuries, and Risk Factors Study (GBD) 2016.

**Methods:**

The motor neuron diseases included in this study were amyotrophic lateral sclerosis, spinal muscular atrophy, hereditary spastic paraplegia, primary lateral sclerosis, progressive muscular atrophy, and pseudobulbar palsy. Incidence, prevalence, and DALYs were estimated using a Bayesian meta-regression model. We analysed 14 165 site-years of vital registration cause of death data using the GBD 2016 cause of death ensemble model. The 84 risk factors quantified in GBD 2016 were tested for an association with incidence or death from motor neuron diseases. We also explored the relationship between Socio-demographic Index (SDI; a compound measure of income per capita, education, and fertility) and age-standardised DALYs of motor neuron diseases.

**Findings:**

In 2016, globally, 330 918 (95% uncertainty interval [UI] 299 522–367 254) individuals had a motor neuron disease. Motor neuron diseases have caused 926 090 (881 566–961 758) DALYs and 34 325 (33 051–35 364) deaths in 2016. The worldwide all-age prevalence was 4·5 (4·1–5·0) per 100 000 people, with an increase in age-standardised prevalence of 4·5% (3·4–5·7) over the study period. The all-age incidence was 0·78 (95% UI 0·71–0·86) per 100 000 person-years. No risk factor analysed in GBD showed an association with motor neuron disease incidence. The largest age-standardised prevalence was in high SDI regions: high-income North America (16·8, 95% UI 15·8–16·9), Australasia (14·7, 13·5–16·1), and western Europe (12·9, 11·7–14·1). However, the prevalence and incidence were lower than expected based on SDI in high-income Asia Pacific.

**Interpretation:**

Motor neuron diseases have low prevalence and incidence, but cause severe disability with a high fatality rate. Incidence of motor neuron diseases has geographical heterogeneity, which is not explained by any risk factors quantified in GBD, suggesting other unmeasured risk factors might have a role. Between 1990 and 2016, the burden of motor neuron diseases has increased substantially. The estimates presented here, as well as future estimates based on data from a greater number of countries, will be important in the planning of services for people with motor neuron diseases worldwide.

**Funding:**

Bill & Melinda Gates Foundation.

## Introduction

Motor neuron diseases are a group of neurodegenerative disorders related to upper and lower motor neuron degeneration, including amyotrophic lateral sclerosis, spinal muscular atrophy, hereditary spastic paraplegia, primary lateral sclerosis, progressive muscular atrophy, and pseudobulbar palsy. Amyotrophic lateral sclerosis, the most common motor neuron disease, is clinically characterised by extensive paralysis leading to death generally by respiratory failure, with 50% of patients dying within 15–20 months after diagnosis.^1^, ^2^

Although spinal muscular atrophy and hereditary spastic paraplegia are known to have a genetic basis, the causes of other motor neuron diseases remain unknown, but are postulated to combine environmental and genetic factors.^1^, ^3^, ^4^ Genetic variants have been associated with amyotrophic lateral sclerosis,^3^ whereas the contribution of environmental factors, with the possible exception of smoking,^5^, ^6^ is still not clear because of the difficulties in assessment of potential risk factors retrospectively in case-control studies.

Previous reviews and meta-analyses using a worldwide approach to study amyotrophic lateral sclerosis reported a median prevalence of 4·48 per 100 000 (IQR 3·03–6·70)^7^ and a standardised incidence rate of 1·68 per 100 000 person-years (95% CI 1·50–1·85)^8^ that varied with geography, sex, and age.^8^ Amyotrophic lateral sclerosis is rare before age 50 years, with peak incidence at age 70 years followed by a sharp decrease in incidence.^8^, ^9^ Previous syntheses of motor neuron disease prevalence,^7^ incidence,^8^ and phenotype^2^ based on population-based studies have had scarce geographical coverage. Although insights into our understanding of the epidemiology of motor neuron diseases, and amyotrophic lateral sclerosis in particular, have been provided by population-based registries in Europe over the past two decades^10^, ^11^ and in the USA more recently,^12^ these epidemiological surveillance systems are not available in other parts of the world. Additionally, worldwide data on other metrics of motor neuron disease burden (eg, mortality, years of life lost [YLLs], years of life lived with disability [YLDs], and disability-adjusted life-years [DALYs]) are still scarce.

**Research in context**

**Evidence before this study**

Motor neuron diseases are a group of rare neurodegenerative disorders (amyotrophic lateral sclerosis, spinal muscular atrophy, hereditary spastic paraplegia, primary lateral sclerosis, progressive muscular atrophy, and pseudobulbar palsy) that are fatal in 50% of affected people within 15 to 20 months after diagnosis. Previous epidemiological studies of motor neuron diseases showed large geographical variations in incidence, but no data on motor neuron disease incidence existed for most countries. We did a systematic review of prevalence, incidence, and mortality risk in PubMed for articles published in English from Jan 1, 1990, to Dec 31, 2015. In the Global Burden of Diseases, Injuries, and Risk Factors Study (GBD), systematic reviews for motor neuron diseases are done every 3 years, with new sources suggested by GBD collaborators included in each round of estimates. The search terms were: ((“motor neuron disease”[MeSH Terms] OR (“motor”[All Fields] AND “neuron”[All Fields] AND “disease”[All Fields]) OR “motor neuron disease”[All Fields] OR (“motor”[All Fields] AND “neuron”[All Fields] AND “diseases”[All Fields]) OR “motor neuron diseases”[All Fields]) OR (“amyotrophic lateral sclerosis”[MeSH Terms] OR (“amyotrophic”[All Fields] AND “lateral“[All Fields] AND “sclerosis”[All Fields]) OR “amyotrophic lateral sclerosis”[All Fields]) OR “ALS”[All Fields] OR (“motor neuron disease”[MeSH Terms] OR (“motor“[All Fields] AND “neuron”[All Fields] AND “disease”[All Fields]) OR “motor neuron disease”[All Fields] OR (“primary”[All Fields] AND “lateral”[All Fields] AND “sclerosis”[All Fields]) OR “primary lateral sclerosis”[All Fields]) OR (“PLS”[Journal] OR “pls”[All Fields]) OR (“muscular atrophy, spinal”[MeSH Terms] OR (“muscular”[All Fields] AND “atrophy”[All Fields] AND “spinal”[All Fields]) OR “spinal muscular atrophy”[All Fields] OR (“progressive”[All Fields] AND “muscular”[All Fields] AND “atrophy”[All Fields]) OR “progressive muscular atrophy”[All Fields]) OR “PBP”[All Fields] OR (“pseudobulbar palsy”[MeSH Terms] OR (“pseudobulbar”[All Fields] AND “palsy”[All Fields]) OR “pseudobulbar palsy”[All Fields])) AND ((“epidemiology”[Subheading] OR “epidemiology”[All Fields] OR “epidemiology”[MeSH Terms]) OR “population-based”[All Fields]).

**Added value of this study**

This is the first concerted effort to make the methods and results of GBD accessible to clinicians and researchers with an interest in motor neuron disease. We show that the burden of motor neuron disease is highest in countries with a high Socio-demographic Index (SDI; a measure of country social development based on education, income, and fertility), especially high-income North America, western Europe, and Australasia, but not in high-income Asia Pacific. We show that age-standardised incidence rates of motor neuron diseases vary within each SDI level, suggesting that causative factors other than sociodemographic development are responsible for the geographical variation in disease occurrence. We also show that the geographical heterogeneity of motor neuron disease burden is not explained by any of the 84 risk factors quantified in GBD, suggesting a role of unmeasured risk factors.

**Implication of all the available evidence**

The burden of motor neuron diseases is currently greatest in high-income countries and this burden is increasing because of population ageing. Small increases in age-standardised incidence and prevalence rates occurred between 1990 and 2016, but we report larger increases in counts because of population ageing and population growth. Despite the absence of a cure for motor neuron diseases, these findings are important to health service planning as the care of patients with motor neuron disease is intensive and expensive. Additional research into the causes of motor neuron disease is needed.

Within the Global Burden of Diseases, Injuries, and Risk Factors Study (GBD) 2016, we quantified the burden of motor neuron diseases in terms of incidence, prevalence, mortality, and DALYs (a sum of YLLs and YLDs) between 1990 and 2016 by age, sex, year, and geographical location. Our study considers motor neuron diseases across the whole age spectrum including spinal muscular atrophy, a rare disorder in the first years of life affecting proximal limbs and respiratory muscles. The relationships between motor neuron disease burden and country development level, as measured by the Socio-demographic Index (SDI), a composite measure of income per capita, years of schooling, and total fertility rate, were also analysed.

## Methods

### Overview

General methods used in GBD 2016 are reported in the appendix. Specific methods relevant to the estimation of motor neuron disease burden are described here. Motor neuron diseases included in this analysis were amyotrophic lateral sclerosis, spinal muscular atrophy, hereditary spastic paraplegia, primary lateral sclerosis, progressive muscular atrophy, and pseudobulbar palsy. We analysed motor neuron diseases across all ages, and included motor neuron disease with a strong genetic component with high prevalence in the early years of life.

### Mortality

The International Classification of Diseases ninth edition (ICD-9) code for motor neuron diseases is 335 and the ICD-10 code is G12. Mortality from motor neuron diseases was modelled by use of CODEm, the cause of death ensemble model used throughout GBD. The model used 14 165 site-years of data—ie, a combination of location and calendar year—as well as predictive covariates on asbestos production, mean serum cholesterol, fruit consumption, average latitude, proportion of the population with access to sanitation, proportion of the population with access to clean water, health-care access and quality,^13^ education, log-transformed lag-distributed income, and SDI.^14^ Predictive covariates in our cause of death analytical tool, CODEm, are based on literature review of factors that have been found to be associated with a disease of interest without necessarily having sufficient evidence for a causal relationship. CODEm is designed to choose the set of covariates that best predicts mortality rates given the available data from vital registrations and verbal autopsy studies.

### Non-fatal disease modelling

The El Escorial Criteria^15^ were used as the reference diagnostic criteria for amyotrophic lateral sclerosis. Spinal muscular atrophy cases defined by ICD codes were also included. Data from administrative health records reporting by ICD codes were also included. We identified 55 sources through our systematic literature review, including 47 sources on incidence, covering seven of the 21 world regions, and eight sources on prevalence, covering five of the 21 world regions (appendix). We also added 3 years (2000, 2010, and 2012) of US medical claims data. There were no data for southeast Asia, Oceania, central Asia, eastern Europe, Australasia, the Caribbean, Andean Latin America, central Latin America, south Asia, central sub-Saharan Africa, southern sub-Saharan Africa, and western sub-Saharan Africa. Where data points spanned age ranges greater than 20 years, we split the data into 5-year age bands using the age pattern of the USA, where we had the most detailed motor neuron disease data by age.

We used DisMod-MR 2.1, the Bayesian meta-regression modelling tool developed for GBD, to generate results that were consistent with the available data on prevalence and incidence, as well as with our estimates for mortality due to motor neuron diseases. In the model, we included a setting of no remission (ie, no cure). We used DisMod-MR 2.1 to adjust for systematic bias in the first 2 years of US claims data, because the data for 2000 and 2010 were systematically lower than the data for 2012. Additionally, we included the absolute value of latitude, as a country-level predictor on prevalence, and included national income, with a built-in short lag, as a country-level predictor on excess mortality rate.

### Motor neuron disease severity and YLDs

To identify the different health consequences of motor neuron diseases, we analysed data from the largest amyotrophic lateral sclerosis clinical dataset, with a total of 8635 patient records: the Pooled Resource Open-access ALS Clinical Trials (PRO-ACT).^16^ These data were compiled by the PRO-ACT Consortium, which is a collaboration between Prize4Life and the Northeast ALS Consortium, with funding from the ALS Therapy Alliance. The data available in the PRO-ACT database have been volunteered by PRO-ACT Consortium members.^17^ We used records with complete information on the Amyotrophic Lateral Sclerosis Functional Rating Scale Revised (ALSFRS-R; n=4838) and selected only the observation at enrolment into a trial to eliminate potential bias from treatment effects.

We used the ALSFRS-R to establish the severity distribution of three symptom domains: motor impairment, respiratory problems, and speech impairment. Motor impairment of the legs was assessed by two questions, and we mapped a combined score of 8 to a state of no impairment, 5–7 to mild impairment, 2–4 to moderate impairment, and 0–1 to severe impairment. Three additional questions assessed fine motor impairment, and a combined score of 12 corresponded to no impairment, 9–11 to mild impairment, 3–8 to moderate impairment, and 0–2 to severe impairment. To summarise total motor impairment, we took the more severe of the two rankings. Respiratory problems were assessed by one question, and a score of 4 indicated no impairment, 3 indicated mild impairment, 2 indicated moderate impairment, and 0–1 indicated severe impairment. Finally, there was one question on speech impairment, and we mapped a score of 4 to no impairment, and any other score to an impaired state.

After establishing the severity status, we estimated the relative proportions of each of the 29 combinations of the different levels on the three domains. To derive combined disability weights for each impairment combination, which constitute the different potential health outcomes of motor neuron disease, we used a multiplicative aggregation formula.^18^

### DALYs

The proportions from the ALSFRS-R analysis were applied to the prevalence estimates derived from DisMod-MR 2.1, and the prevalence of each long-term outcome was multiplied by the combined disability weights to get uncorrected YLDs. The initial YLD estimates were adjusted for comorbidity by use of the comorbidity simulation across all causes included in GBD 2016.^19^ We calculated YLLs by multiplying the number of deaths in each age group by the remaining life expectancy derived from the GBD standard life table (appendix). DALYs were then calculated by summing YLLs and YLDs.

Uncertainty was propagated by sampling 1000 draws at each computational step, which allowed for the combination of uncertainty from different sources, including input data, data processing steps, and residual non-sampling error. Uncertainty intervals (UIs) were defined as the 25th and 975th values of the ordered draws and were used to define significant change over time. This study complies with the Guidelines for Accurate and Transparent Health Estimates Reporting (GATHER) recommendations (appendix).

### Risk factors

In GBD 2016, the contribution of 84 risks and combinations of risks to disease burden was quantified.^20^ Criteria for inclusion of risks into GBD include: the availability of sufficient evidence for a causal relationship between a risk and one or more disease or injury outcome; evidence to support generalisability of an effect size beyond the populations included in epidemiological studies; availability of sufficient data and methods to enable estimation of exposure levels by country; and the probable importance of a risk factor to disease burden or policy considerations.^20^

### Role of the funding source

The funder of the study had no role in study design, data collection, data analysis, data interpretation, or the writing of the report. All authors had full access to the data in the study and had final responsibility for the decision to submit for publication.

## Results

All results presented in this paper can be found in the GBD 2016 online results tool, with all estimates from 1990 to 2016 for all world regions. The same results can also be explored by use of the GBD 2016 data visualisation tool.

In 2016, 330 918 (95% UI 299 522–367 254) individuals worldwide had a motor neuron disease: 161 901 (48·9%) in high SDI, 50 286 (15·2%) in high-middle SDI, 70 924 (21·4%) in middle SDI, 38 873 (11·8%) in low-middle SDI, and 8545 (2·6%) in low SDI countries (table). The all-age global prevalence was 4·5 (4·1–5·0) per 100 000 population. Globally, we estimated there to be 57 452 (52 522–63 242) incident cases of a motor neuron disease, and all-age global incidence of motor neuron disease to be 0·78 (0·71–0·86) per 100 000 person-years. We estimated there to be 34 325 (33 051–35 364) motor neuron disease-related deaths (table), with an all-age cause-specific mortality rate of 0·46 (0·45–0·48) per 100 000 person-years. Motor neuron diseases caused 926 090 (881 566–961 758) DALYs (table; age-standardised DALYs rate 13·2, 12·5–13·7), of which 70 165 (49 321–93 203) were YLDs (age-standardised rate 1·0, 0·7–1·3) and 855 924 (819 373–883 289) were YLLs (age-standardised rate 12·2, 11·6–12·6).

**Table:** Deaths, prevalence, and DALYs for motor neuron disease in 2016 and percentage change of age standardised rates by location

	**Deaths**		**Prevalence**		**DALYs**	
	2016 counts	Percentage change in age-standardised rates, 1990–2016	2016 counts	Percentage change in age-standardised rates, 1990–2016	2016 counts	Percentage change in age-standardised rates, 1990–2016
**Global**	**34 325 (33 051 to 35 364)**	**8·0% (−0·1 to 12·2)**	**330 918 (299 522 to 367 254)**	**4·5% (3·4 to 5·7)**	**926 090 (881 566 to 961 758)**	**−1·5% (−9·3 to 2·9)**
High SDI	21 162 (20 081 to 21 830)	14·1% (5·2 to 19·1)	161 901 (150 024 to 174 346)	19·7% (18·0 to 21·6)	479 748 (455 714 to 496 340)	3·8% (−3·7 to 8·2)
High-middle SDI	4712 (4392 to 4972)	20·7% (2·9 to 31·8)	50 286 (44 798 to 56 819)	16·2% (14·2 to 17·9)	144 696 (134 507 to 153 708)	0·4% (−19·2 to 17·9)
Middle SDI	5183 (4934 to 5387)	22·7% (9·7 to 30·6)	70 924 (61 566 to 82 419)	12·3% (10·3 to 14·0)	177 160 (166 987 to 185 970)	5·7% (−6·0 to 18·0)
Low-middle SDI	2813 (2455 to 3304)	36·7% (11·7 to 57·3)	38 873 (33 761 to 45 357)	10·2% (8·9 to 11·4)	104 268 (93 547 to 119 177)	21·6% (4·8 to 37·3)
Low SDI	443 (382 to 648)	20·2% (−1·8 to 41·9)	8545 (7405 to 10 007)	3·8% (2·8 to 5·0)	19 786 (17 506 to 26 045)	7·1% (−12·9 to 31·5)
**High-income North America**	**8592 (8226 to 8870)**	**27·2% (19·7 to 32·8)**	**72 577 (68 184 to 77 282)**	**7·4% (4·4 to 10·8)**	**204 447 (195 872 to 212 530)**	**14·1% (8·0 to 18·9)**
Canada	959 (873 to 1045)	20·2% (6·8 to 33·6)	10 013 (9208 to 10 952)	42·1% (35·4 to 49·3)	22 072 (20 194 to 23 900)	12·7% (1·0 to 24·3)
Greenland	0 (0 to 1)	3·3% (−24·6 to 41·1)	8 (7 to 9)	29·8% (25·1 to 35·0)	11 (9 to 14)	−1·4% (−24·6 to 27·5)
USA	7632 (7315 to 7902)	27·9% (20·3 to 34·0)	62 531 (58 894 to 66 406)	3·4% (0·1 to 7·2)	182 359 (174 927 to 189 911)	14·3% (8·1 to 19·3)
**Australasia**	**850 (776 to 925)**	**20·0% (6·9 to 32·3)**	**5065 (4632 to 5506)**	**29·4% (25·1 to 33·9)**	**18 723 (17 236 to 20 191)**	**11·3% (0·0 to 23·1)**
Australia	711 (643 to 779)	21·9% (6·8 to 36·3)	4247 (3886 to 4621)	30·3% (25·4 to 35·4)	15 625 (14 212 to 17 040)	12·7% (−1·1 to 26·1)
New Zealand	139 (122 to 157)	10·8% (−5·6 to 26·5)	818 (747 to 887)	25·1% (19·8 to 30·8)	3097 (2730 to 3465)	3·5% (−10·8 to 18·1)
**High-income Asia-Pacific**	**1919 (1777 to 2055)**	**−9·2% (−15·6 to −2·9)**	**17 574 (15 713 to 19 782)**	**18·9% (16·5 to 21·5)**	**40 294 (37 320 to 43 001)**	**−18·1% (−23·4 to −12·8)**
Brunei	1 (0 to 1)	7·3% (−24·0 to 41·3)	19 (17 to 21)	16·2% (12·2 to 20·9)	23 (18 to 28)	7·3% (−20·7 to 38·8)
Japan	1796 (1660 to 1927)	−2·5% (−10·2 to 4·5)	13 893 (12 394 to 15 659)	19·9% (17·6 to 22·5)	36 101 (33 402 to 38 439)	−11·9% (−18·2 to −6·6)
Singapore	23 (19 to 29)	9·9% (−14·2 to 39·2)	243 (219 to 271)	20·6% (16·0 to 25·1)	718 (592 to 868)	−1·2% (−21·0 to 24·5)
South Korea	100 (72 to 130)	−33·9% (−55·6 to −8·1)	3419 (3033 to 3860)	26·3% (21·5 to 31·3)	3452 (2658 to 4327)	−34·7% (−52·4 to −12·0)
**Western Europe**	**10 357 (9723 to 10 818)**	**15·2% (3·6 to 22·1)**	**71 288 (65 330 to 77 548)**	**31·2% (29·1 to 33·3)**	**227 135 (213 738 to 237 121)**	**−0·2% (−9·8 to 6·1)**
Andorra	2 (2 to 3)	13·2% (−15·4 to 52·2)	15 (14 to 17)	21·0% (16·6 to 25·3)	50 (36 to 62)	−0·4% (−23·2 to 30·2)
Austria	152 (136 to 168)	36·8% (20·2 to 54·1)	1282 (1172 to 1405)	36·9% (31·6 to 42·3)	3721 (3367 to 4114)	17·2% (1·0 to 34·7)
Belgium	263 (232 to 293)	24·8% (6·5 to 41·6)	1850 (1699 to 2019)	37·2% (31·8 to 43·4)	5983 (5353 to 6648)	10·9% (−4·9 to 26·9)
Cyprus	13 (11 to 14)	−15·4% (−47·2 to 4·2)	108 (98 to 118)	19·7% (14·8 to 24·8)	303 (273 to 336)	−27·4% (−55·4 to −5·5)
Denmark	134 (114 to 156)	−15·4% (−29·5 to 0·4)	983 (899 to 1078)	20·8% (16·5 to 25·9)	2915 (2487 to 3339)	−19·4% (−31·6 to −5·5)
Finland	183 (159 to 204)	−23·8% (−38·5 to −9·1)	1207 (1103 to 1319)	19·5% (14·4 to 25·2)	3959 (3455 to 4405)	−24·2% (−38·4 to −11·3)
France	1557 (1408 to 1707)	5·0% (−7·5 to 17·7)	11 712 (10 762 to 12 638)	27·3% (22·5 to 32·1)	34 427 (31 349 to 37 585)	−6·1% (−17·5 to 5·6)
Germany	1911 (1698 to 2129)	1·3% (−16·3 to 19·9)	12 502 (11 442 to 13 684)	27·5% (22·7 to 32·9)	42 111 (37 875 to 46 516)	−9·9% (−26·6 to 6·6)
Greece	111 (96 to 124)	156·3% (104·4 to 202·3)	1141 (1031 to 1256)	29·4% (24·2 to 34·6)	2994 (2620 to 3375)	114·9% (51·4 to 166·4)
Iceland	8 (7 to 9)	23·7% (5·9 to 41·6)	61 (56 to 67)	25·1% (20·2 to 30·2)	188 (169 to 208)	12·8% (−2·6 to 28·6)
Ireland	120 (104 to 138)	24·7% (4·4 to 47·8)	835 (764 to 911)	37·6% (31·6 to 44·0)	2927 (2571 to 3344)	17·9% (−1·5 to 38·5)
Israel	73 (59 to 89)	−23·2% (−43·1 to −0·9)	784 (714 to 863)	17·4% (12·7 to 22·1)	1991 (1642 to 2373)	−29·6% (−47·2 to −9·8)
Italy	1418 (1 270 to 1 570)	35·8% (18·6 to 54·5)	9553 (8745 to 10 416)	34·5% (28·4 to 40·4)	30 525 (27 281 to 33 409)	9·0% (−6·5 to 26·1)
Luxembourg	9 (8 to 10)	4·4% (−11·4 to 20·3)	86 (79 to 95)	27·2% (22·1 to 32·9)	217 (192 to 245)	−7·5% (−21·3 to 6·4)
Malta	8 (7 to 10)	14·5% (−5·9 to 40·8)	62 (56 to 67)	32·2% (26·7 to 37·9)	194 (164 to 233)	15·5% (−4·0 to 38·4)
Netherlands	494 (443 to 554)	7·8% (−7·1 to 22·8)	3347 (3060 to 3648)	28·1% (23·0 to 33·1)	11 110 (9988 to 12 405)	−1·4% (−14·5 to 11·8)
Norway	124 (107 to 141)	1·9% (−13·2 to 18·0)	1046 (961 to 1153)	28·0% (22·3 to 33·5)	2764 (2416 to 3125)	−8·4% (−21·2 to 5·3)
Portugal	185 (166 to 205)	95·7% (70·3 to 124·7)	1365 (1250 to 1489)	37·6% (31·7 to 43·8)	4639 (4173 to 5106)	48·2% (21·7 to 73·4)
Spain	905 (819 to 991)	41·1% (25·9 to 56·8)	6436 (5888 to 7035)	36·7% (31·0 to 42·3)	20 680 (18 811 to 22 572)	12·1% (−1·0 to 26·0)
Sweden	304 (266 to 344)	35·9% (15·6 to 55·6)	2153 (1944 to 2416)	33·5% (28·7 to 39·3)	6141 (5401 to 897)	25·3% (7·8 to 43·3)
Switzerland	184 (143 to 233)	−7·4% (−31·0 to 22·2)	1441 (1314 to 1578)	22·8% (17·9 to 28·3)	3971 (3164 to 4972)	−12·9% (−33·9 to 12·7)
United Kingdom	2199 (2075 to 2281)	23·8% (15·5 to 29·3)	13 249 (12 000 to 14 556)	34·5% (31·7 to 37·8)	45 310 (42 802 to 47 187)	0·2% (−6·1 to 4·5)
**Southern Latin America**	**477 (423 to 532)**	**17·9% (−4·4 to 36·0)**	**4527 (4103 to 5021)**	**27·8% (24·7 to 31·1)**	**12 806 (11 433 to 14 281)**	**12·6% (−8·3 to 29·1)**
Argentina	279 (247 to 309)	29·2% (4·0 to 48·4)	2850 (2576 to 3164)	27·8% (23·6 to 32·5)	7570 (6748 to 8342)	22·0% (−0·3 to 38·8)
Chile	152 (116 to 192)	4·7% (−26·3 to 41·0)	1391 (1253 to 1550)	27·6% (23·3 to 32·1)	4123 (3177 to 5265)	0·2% (−28·0 to 33·8)
Uruguay	47 (42 to 51)	−1·5% (−15·5 to 12·7)	285 (261 to 314)	23·4% (18·9 to 28·3)	1112 (998 to 1217)	−4·2% (−17·5 to 8·2)
**Eastern Europe**	**729 (583 to 909)**	**36·1% (6·0 to 72·7)**	**6497 (5784 to 7341)**	**3·2% (0·3 to 6·0)**	**21 974 (17 757 to 27 107)**	**24·3% (−0·7 to 54·7)**
Belarus	33 (26 to 39)	29·3% (4·2 to 56·1)	290 (257 to 329)	5·4% (1·3 to 9·5)	934 (757 to 1 121)	17·7% (−3·3 to 42·2)
Estonia	6 (5 to 7)	−1·0% (−16·8 to 14·6)	45 (41 to 51)	2·7% (−0·6 to 6·5)	156 (133 to 177)	−12·5% (−26·0 to 1·6)
Latvia	10 (9 to 12)	114·3% (75·3 to 151·7)	66 (59 to 74)	7·0% (3·3 to 10·7)	273 (230 to 312)	85·2% (50·8 to 116·4)
Lithuania	30 (26 to 33)	133·8% (99·8 to 167·1)	121 (110 to 135)	21·3% (15·7 to 26·8)	743 (658 to 824)	105·3% (76·2 to 133·9)
Moldova	9 (8 to 11)	16·9% (−5·7 to 41·9)	100 (88 to 114)	1·1% (−2·4 to 5·1)	297 (254 to 345)	15·9% (−5·4 to 38·2)
Russia	486 (357 to 655)	39·9% (−0·3 to 90·8)	4 587 (4 079 to 5 196)	2·7% (−1·2 to 6·8)	15 218 (11 485 to 20 363)	25·3% (−7·0 to 65·5)
Ukraine	155 (120 to 199)	18·5% (−10·5 to 57·9)	1287 (1141 to 1443)	1·8% (−2·4 to 5·9)	4352 (3386 to 5553**)**	14·0% (−11·8 to 48·9)
**Central Europe**	**691 (636 to 742)**	**5·3% (−9·7 to 15·7)**	**4449 (3998 to 4974)**	**4·2% (2·1 to 6·5)**	**19 034 (17 601 to 20 320)**	**−9·3% (−20·9 to −0·3)**
Albania	6 (5 to 7)	−7·2% (−24·2 to 11·7)	88 (78 to 99)	6·8% (2·5 to 10·9)	192 (161 to 221)	−15·0% (−30·7 to 1·4)
Bosnia and Herzegovina	13 (11 to 15)	22·5% (−18·2 to 57·7)	128 (114 to 145)	10·1% (5·6 to 14·8)	373 (317 to 439)	16·6% (−19·6 to 46·4)
Bulgaria	17 (14 to 20)	−28·5% (−42·5 to −13·1)	245 (216 to 277)	−4·5% (−9·1 to 0·1)	493 (414 to 580)	−40·9% (−51·5 to −28·6)
Croatia	38 (32 to 44)	12·1% (−5·7 to 32·3)	179 (161 to 198)	−0·4% (−5·0 to 4·8)	1054 (907 to 1202)	−6·9% (−21·6 to 10·3)
Czech Republic	77 (69 to 85)	0·5% (−12·9 to 13·8)	443 (397 to 496)	−0·8% (−5·1 to 3·8)	2101 (1893 to 2306)	−16·1% (−28·7 to −2·1)
Hungary	80 (70 to 90)	0·3% (−14·5 to 16·7)	393 (353 to 438)	−1·5% (−6·1 to 3·5)	2289 (2013 to 2578)	−18·6% (−32·2 to −1·4)
Macedonia	4 (4 to 5)	−52·0% (−62·5 to −40·1)	64 (57 to 72)	−5·8% (−10·4 to −0·8)	136 (121 to 151)	−70·1% (−78·5 to −58·3)
Montenegro	1 (1 to 1)	3·0% (−14·6 to 23·0)	20 (18 to 23)	4·1% (−0·1 to 8·3)	39 (33 to 44)	−5·3% (−19·7 to 11·8)
Poland	346 (307 to 389)	2·9% (−18·9 to 21·2)	1695 (1528 to 1888)	6·5% (2·4 to 10·9)	9135 (8111 to 10 229)	−8·7% (−28·0 to 7·5)
Romania	61 (54 to 68)	24·4% (1·4 to 47·1)	639 (566 to 720)	6·4% (2·2 to 10·7)	1825 (1623 to 2047)	8·5% (−12·3 to 29·5)
Serbia	23 (20 to 25)	22·5% (3·9 to 43·3)	288 (256 to 327)	3·9% (−0·5 to 8·3)	662 (595 to 727)	8·9% (−7·2 to 26·0)
Slovakia	21 (17 to 24)	42·5% (13·7 to 69·6)	192 (171 to 217)	6·1% (2·1 to 10·2)	611 (512 to 704)	25·0% (−0·2 to 50·2)
Slovenia	4 (4 to 5)	−79·2% (−82·8 to −74·5)	77 (68 to 87)	−20·1% (−25·4 to −14·4)	124 (106 to 142)	−82·4% (−85·6 to −78·3)
**Central Asia**	**114 (100 to 130)**	**5·3% (−5·8 to 20·0)**	**1830 (1612 to 2083)**	**2·3% (0·6 to 3·9)**	**4127 (3637 to 4706)**	**−0·5% (−10·0 to 10·0)**
Armenia	5 (4 to 5)	5·8% (−12·2 to 24·4)	73 (65 to 83)	3·7% (−0·4 to 7·3)	149 (131 to 168)	−2·8% (−17·5 to 13·1)
Azerbaijan	13 (11 to 16)	35·7% (0·5 to 101·2)	215 (190 to 246)	5·2% (1·6 to 9·2)	477 (390 to 575)	24·2% (−5·1 to 78·2)
Georgia	9 (8 to 11)	−6·4% (−26·9 to 21·4)	102 (90 to 115)	1·7% (−1·8 to 5·4)	290 (238 to 353)	−6·3% (−25·9 to 19·9)
Kazakhstan	34 (23 to 47)	−2·2% (−21·6 to 22·5)	417 (366 to 474)	4·0% (0·2 to 8·0)	1180 (836 to 1634)	−6·0% (−23·3 to 15·4)
Kyrgyzstan	6 (6 to 7)	3·2% (−16·7 to 30·3)	113 (99 to 129)	−0·1% (−3·7 to 3·5)	260 (226 to 293)	1·8% (−17·6 to 27·3)
Mongolia	3 (3 to 4)	38·4% (0·4 to 76·2)	60 (53 to 69)	6·8% (3·1 to 11·0)	121 (102 to 142)	21·2% (−3·5 to 47·0)
Tajikistan	5 (4 to 8)	−1·9% (−23·8 to 25·0)	146 (127 to 169)	−1·1% (−4·5 to 2·2)	245 (196 to 320)	−13·4% (−34·8 to 10·9)
Turkmenistan	8 (6 to 10)	23·9% (−3·1 to 46·9)	107 (94 to 122)	5·7% (1·8 to 9·3)	314 (245 to 380)	11·8% (−12·9 to 33·1)
Uzbekistan	30 (26 to 34)	37·2% (4·7 to 73·7)	597 (524 to 682)	4·0% (0·2 to 8·0)	1091 (949 to 1249)	25·9% (−0·4 to 55·0)
**Central Latin America**	**748 (683 to 791)**	**57·0% (39·4 to 67·7)**	**7609 (6627 to 8760)**	**9·7% (8·3 to 11·1)**	**23 660 (21 696 to 25 181)**	**37·1% (23·0 to 45·8)**
Colombia	176 (150 to 201)	67·9% (44·4 to 93·6)	1383 (1233 to 1564)	12·4% (8·2 to 16·6)	5306 (4562 to 6054)	41·7% (21·2 to 62·0)
Costa Rica	41 (35 to 46)	47·6% (23·6 to 73·3)	209 (189 to 232)	17·2% (12·5 to 22·2)	1093 (940 to 1239)	48·6% (24·4 to 73·2)
El Salvador	9 (8 to 11)	19·9% (−4·6 to 46·9)	157 (139 to 180)	7·7% (4·0 to 11·8)	296 (250 to 342)	11·9% (−9·1 to 34·6)
Guatemala	16 (12 to 20)	26·6% (−14·9 to 113·6)	367 (319 to 423)	5·7% (2·0 to 9·5)	570 (449 to 717)	19·4% (−16·4 to 88·2)
Honduras	14 (11 to 20)	43·2% (−0·2 to 99·3)	192 (168 to 220)	6·3% (2·4 to 10·5)	501 (375 to 660)	25·5% (−11·0 to 70·5)
Mexico	381 (351 to 405)	64·2% (51·7 to 74·1)	4187 (3577 to 4949)	9·8% (8·1 to 11·7)	12 494 (11 556 to 13 234)	38·7% (28·5 to 46·7)
Nicaragua	8 (6 to 9)	33·6% (−1·3 to 73·1)	148 (130 to 168)	4·2% (0·6 to 8·0)	266 (219 to 316)	20·2% (−6·6 to 51·1)
Panama	12 (10 to 15)	9·8% (−18·0 to 37·0)	117 (104 to 131)	9·4% (5·6 to 13·0)	384 (312 to 450)	9·4% (−16·8 to 35·7)
Venezuela	90 (74 to 108)	28·6% (−4·7 to 67·0)	850 (751 to 966)	5·1% (1·6 to 9·0)	2750 (2235 to 3288)	27·5% (−5·0 to 65·5)
**Andean Latin America**	**114 (98 to 130)**	**8·3% (−9·9 to 26·0)**	**1408 (1249 to 1596)**	**6·5% (4·3 to 9·0)**	**3564 (3085 to 4013)**	**−1·7% (−18·7 to 14·6)**
Bolivia	26 (19 to 34)	24·1% (−28·0 to 72·0)	246 (216 to 281)	7·6% (3·9 to 11·5)	804 (598 to 1047)	11·4% (−36·3 to 51·9)
Ecuador	39 (35 to 44)	60·6% (31·3 to 84·0)	382 (337 to 435)	7·6% (3·8 to 11·0)	1221 (1077 to 1361)	43·5% (16·9 to 63·4)
Peru	49 (39 to 60)	−17·5% (−36·1 to 7·6)	779 (691 to 887)	5·6% (2·3 to 9·3)	1539 (1243 to 1855)	−24·2% (−41·4 to −0·4)
**Caribbean**	**188 (171 to 204)**	**3·6% (−9·5 to 19·9)**	**1481 (1326 to 1657)**	**2·8% (0·8 to 4·9)**	**5721 (5093 to 6327)**	**−5·0% (−20·5 to 10·3)**
Antigua and Barbuda	0 (0 to 0)	−5·7% (−23·1 to 13·6)	3 (3 to 3)	4·9% (1·5 to 8·4)	7 (6 to 8)	−4·7% (−20·7 to 12·7)
The Bahamas	2 (2 to 2)	−5·2% (−28·1 to 31·6)	14 (13 to 16)	−0·2% (−3·8 to 3·3)	59 (51 to 69)	−12·5% (−33·6 to 21·4)
Barbados	4 (3 to 4)	12·7% (−5·7 to 36·5)	14 (13 to 15)	4·3% (0·8 to 7·8)	95 (84 to 107)	12·0% (−6·5 to 37·2)
Belize	1 (1 to 1)	136·6% (46·9 to 229·1)	10 (9 to 11)	9·8% (5·6 to 13·6)	33 (25 to 43)	89·1% (23·8 to 167·1)
Bermuda	0 (0 to 0)	−36·5% (−53·5 to −13·5)	3 (3 to 4)	−5·4% (−8·9 to −1·9)	11 (8 to 13)	−41·5% (−55·6 to −18·2)
Cuba	91 (81 to 101)	−2·6% (−17·8 to 18·5)	502 (449 to 559)	2·7% (−1·3 to 6·8)	2500 (2228 to 2769)	−11·1% (−24·1 to 6·7)
Dominica	1 (0 to 1)	50·4% (4·7 to 88·4)	3 (2 to 3)	13·8% (10·1 to 18·4)	16 (14 to 19)	51·9% (7·4 to 89·6)
Dominican Republic	16 (13 to 20)	13·7% (−23·7 to 45·7)	303 (268 to 344)	8·5% (4·8 to 12·6)	529 (428 to 631)	0·3% (−30·0 to 26·3)
Grenada	1 (0 to 1)	72·9% (20·0 to 148·8)	3 (3 to 4)	13·6% (9·1 to 18·0)	16 (13 to 19)	64·5% (16·1 to 133·6)
Guyana	2 (2 to 3)	−1·5% (−22·9 to 20·6)	18 (16 to 21)	9·0% (5·1 to 13·3)	72 (59 to 86)	−8·5% (−27·5 to 12·4)
Haiti	23 (16 to 31)	16·6% (−31·3 to 62·3)	225 (196 to 260)	2·8% (−0·6 to 6·3)	957 (633 to 1 391)	−1·7% (−45·3 to 45·8)
Jamaica	11 (9 to 14)	45·5% (4·1 to 92·6)	91 (81 to 102)	5·8% (1·8 to 9·8)	424 (318 to 568)	31·7% (−12·2 to 87·8)
Puerto Rico	24 (21 to 27)	16·2% (−6·9 to 39·6)	156 (141 to 174)	8·0% (4·1 to 11·5)	636 (555 to 709)	5·7% (−14·7 to 25·7)
Saint Lucia	1 (1 to 1)	61·5% (15·4 to 124·7)	6 (6 to 7)	14·8% (10·8 to 19·2)	33 (30 to 37)	50·8% (8·1 to 106·5)
Saint Vincent and the Grenadines	1 (1 to 1)	176·2% (136·0 to 228·0)	4 (3 to 4)	13·1% (9·1 to 17·0)	19 (17 to 21)	131·0% (86·8 to 178·2)
Suriname	2 (2 to 2)	55·8% (27·8 to 85·3)	15 (13 to 17)	10·0% (6·0 to 14·1)	65 (56 to 75)	39·5% (14·7 to 68·3)
Trinidad and Tobago	7 (6 to 7)	6·5% (−16·4 to 29·3)	46 (41 to 52)	7·8% (4·0 to 12·0)	197 (173 to 221)	10·0% (−13·5 to 35·0)
Virgin Islands	2 (1 to 2)	58·9% (21·5 to 95·1)	6 (6 to 7)	18·1% (13·4 to 23·5)	39 (32 to 46)	45·6% (12·1 to 80·1)
**Tropical Latin America**	**1155 (1073 to 1219)**	**79·1% (60·0 to 90·1)**	**8125 (7084 to 9365)**	**20·0% (17·2 to 23·1)**	**33 632 (31 235 to 35 552)**	**57·7% (40·5 to 68·1)**
Brazil	1132 (1051 to 1197)	77·3% (58·9 to 88·3)	7919 (6904 to 9132)	20·3% (17·4 to 23·5)	32 909 (30 585 to 34 846)	56·2% (39·4 to 66·6)
Paraguay	23 (19 to 26)	215·4% (120·4 to 311·1)	206 (184 to 233)	10·8% (6·8 to 14·9)	723 (610 to 834)	177·6% (101·4 to 253·5)
**East Asia**	**3097 (2956 to 3234)**	**−1·3% (−12·4 to 16·3)**	**56 465 (48 633 to 65 987)**	**18·2% (15·3 to 20·9)**	**111 827 (104 988 to 118 511)**	**−11·6% (−22·7 to 13·2)**
China	2988 (2839 to 3122)	−1·1% (−12·4 to 17·4)	54 405 (46 781 to 63 692)	18·6% (15·6 to 21·4)	107 731 (100 956 to 114 351)	−12·1% (−23·8 to 13·8)
North Korea	60 (43 to 96)	14·6% (−6·1 to 43·8)	955 (847 to 088)	2·6% (−0·9 to 6·0)	2477 (1814 to 3998)	12·6% (−11·9 to 45·5)
Taiwan (province of China)	49 (40 to 57)	−28·0% (−44·3 to −12·3)	1104 (981 to 1253)	13·5% (9·2 to 17·5)	1619 (1354 to 1881)	−22·9% (−38·2 to −8·1)
**Southeast Asia**	**903 (770 to 997)**	**25·2% (0·5 to 37·0)**	**16 378 (14 345 to 18 910)**	**10·4% (9·2 to 11·6)**	**33 240 (28 726 to 36 864)**	**10·5% (−5·9 to 20·4)**
Cambodia	15 (11 to 19)	36·7% (−0·1 to 74·7)	316 (278 to 362)	10·6% (7·0 to 14·3)	622 (457 to 783)	20·6% (−13·0 to 58·5)
Indonesia	335 (232 to 397)	43·1% (24·6 to 59·8)	6151 (5193 to 7307)	10·6% (9·0 to 12·4)	12 963 (9505 to 15 281)	17·0% (2·5 to 38·1)
Laos	7 (5 to 10)	24·9% (−11·7 to 79·0)	142 (124 to 163)	11·1% (7·4 to 15·2)	390 (242 to 652)	10·1% (−28·4 to 70·3)
Malaysia	43 (33 to 49)	23·8% (−17·2 to 54·9)	814 (722 to 925)	8·6% (4·9 to 12·4)	1541 (1236 to 1761)	17·8% (−18·4 to 44·5)
Maldives	0 (0 to 0)	−24·4% (−48·9 to 21·6)	8 (7 to 10)	8·7% (5·3 to 12·5)	13 (11 to 16)	−34·7% (−55·7 to 11·8)
Mauritius	3 (3 to 4)	2·7% (−14·7 to 21·5)	41 (37 to 47)	8·6% (5·4 to 12·9)	103 (89 to 121)	−3·5% (−18·6 to 13·7)
Myanmar	110 (93 to 131)	26·7% (−13·7 to 60·1)	1285 (1139 to 1465)	13·3% (9·3 to 17·4)	3879 (3307 to 4615)	17·6% (−16·1 to 49·6)
Philippines	102 (79 to 124)	17·0% (−11·3 to 44·4)	2309 (2034 to 2655)	6·7% (3·3 to 10·2)	3903 (3142 to 4678)	2·5% (−19·9 to 24·7)
Sri Lanka	40 (32 to 51)	21·6% (−16·3 to 62·9)	593 (523 to 674)	9·6% (6·0 to 13·0)	1360 (1074 to 1677)	−0·2% (−32·6 to 40·2)
Seychelles	0 (0 to 0)	0·6% (−24·5 to 24·7)	3 (2 to 3)	7·5% (4·0 to 11·2)	7 (5 to 8)	−3·8% (−26·8 to 17·2)
Thailand	123 (106 to 142)	−3·4% (−26·3 to 17·7)	2166 (1911 to 2458)	10·0% (6·7 to 13·7)	4091 (3555 to 4724)	−7·3% (−27·7 to 11·9)
Timor–Leste	1 (1 to 1)	39·7% (−12·3 to 136·9)	23 (20 to 26)	13·6% (10·0 to 17·3)	39 (27 to 55)	11·5% (−30·4 to 94·6)
Vietnam	123 (99 to 153)	28·3% (−6·5 to 69·3)	2504 (2209 to 2846)	11·4% (7·8 to 15·6)	4324 (3536 to 5319)	14·0% (−13·6 to 46·4)
**Oceania**	**13 (10 to 18)**	**−5·7% (−21·2 to 12·4)**	**212 (187 to 244)**	**4·0% (1·6 to 6·5)**	**467 (384 to 621)**	**−3·8% (−19·4 to 15·3)**
American Samoa	0 (0 to 0)	−22·1% (−42·3 to −0·3)	2 (2 to 2)	0·0% (−3·6 to 3·8)	5 (4 to 6)	−19·2% (−39·4 to 2·8)
Federated States of Micronesia	0 (0 to 0)	23·7% (−21·4 to 72·2)	2 (2 to 2)	6·4% (2·8 to 10·4)	8 (6 to 11)	22·0% (−19·7 to 71·1)
Fiji	2 (1 to 2)	33·5% (−14·7 to 88·8)	22 (19 to 25)	7·0% (3·5 to 10·5)	52 (40 to 66)	31·3% (−11·1 to 82·3)
Guam	1 (1 to 1)	−45·2% (−56·6 to −30·4)	6 (6 to 7)	−16·3% (−20·2 to −12·5)	30 (24 to 35)	−42·6% (−54·6 to −27·4)
Kiribati	0 (0 to 0)	46·4% (10·2 to 95·3)	2 (2 to 2)	4·7% (1·4 to 8·4)	9 (7 to 12)	46·0% (10·7 to 96·7)
Marshall Islands	0 (0 to 0)	0·9% (−27·8 to 29·8)	1 (1 to 2)	4·7% (0·9 to 8·2)	4 (3 to 6)	1·1% (−25·3 to 28·8)
Northern Mariana Islands	0 (0 to 0)	43·9% (2·4 to 97·0)	3 (3 to 4)	5·5% (2·1 to 9·2)	8 (6 to 11)	30·4% (−6·9 to 78·5)
Papua New Guinea	8 (5 to 12)	−1·3% (−22·4 to 30·4)	135 (118 to 156)	6·9% (3·2 to 10·7)	290 (203 to 424)	−2·6% (−23·6 to 27·6)
Samoa	0 (0 to 1)	−7·6% (−26·6 to 19·1)	5 (4 to 5)	5·8% (2·4 to 9·4)	10 (7 to 17)	−6·6% (−25·4 to 19·0)
Solomon Islands	1 (1 to 1)	6·6% (−17·0 to 44·9)	11 (9 to 13)	5·4% (1·6 to 8·9)	27 (21 to 40)	9·5% (−15·2 to 49·2)
Tonga	0 (0 to 0)	−1·4% (−25·5 to 29·2)	3 (2 to 3)	5·4% (2·2 to 9·1)	6 (5 to 8)	0·1% (−22·7 to 30·8)
Vanuatu	0 (0 to 1)	14·3% (−15·7 to 52·6)	6 (5 to 6)	5·3% (1·7 to 8·9)	15 (12 to 23)	17·2% (−13·3 to 56·3)
**North Africa and Middle East**	**1139 (964 to 1276)**	**5·7% (−23·6 to 24·5)**	**13 291 (11 746 to 15 182)**	**3·1% (1·7 to 4·4)**	**45 475 (38 290 to 51 031)**	**−14·3% (−40·1 to 9·3)**
Afghanistan	37 (21 to 59)	31·8% (4·2 to 71·9)	517 (444 to 603)	3·2% (−0·8 to 7·0)	1689 (945 to 2676)	22·7% (−3·3 to 59·7)
Algeria	60 (51 to 70)	6·9% (−16·4 to 32·5)	943 (832 to 1 079)	2·9% (−1·4 to 7·0)	2332 (1961 to 2736)	−8·6% (−31·9 to 16·7)
Bahrain	1 (1 to 2)	−7·6% (−31·9 to 21·7)	36 (31 to 42)	4·1% (0·4 to 8·3)	60 (47 to 76)	−10·5% (−32·2 to 17·0)
Egypt	168 (102 to 217)	29·6% (−1·6 to 62·3)	1908 (1682 to 2185)	7·1% (3·0 to 11·6)	6211 (3881 to 7959)	7·4% (−16·1 to 33·7)
Iran	106 (86 to 129)	84·9% (19·8 to 175·5)	2012 (1781 to 2283)	7·3% (3·3 to 11·7)	3839 (3181 to 4608)	58·6% (9·7 to 120·1)
Iraq	36 (28 to 46)	25·3% (−13·8 to 72·9)	782 (676 to 907)	3·6% (−0·2 to 7·5)	1684 (1287 to 2256)	17·1% (−16·8 to 60·1)
Jordan	9 (7 to 11)	39·2% (−18·7 to 106·4)	174 (153 to 200)	4·5% (0·5 to 9·0)	431 (338 to 530)	42·3% (−11·0 to 99·7)
Kuwait	5 (4 to 7)	−21·0% (−43·4 to 10·9)	116 (102 to 134)	−0·3% (−4·2 to 3·9)	263 (198 to 346)	−29·3% (−47·6 to −1·7)
Lebanon	8 (5 to 11)	−27·7% (−65·6 to 8·0)	159 (140 to 179)	3·2% (−1·0 to 7·2)	273 (182 to 360)	−32·4% (−66·2 to 0·9)
Libya	15 (8 to 20)	40·9% (−4·5 to 84·2)	150 (132 to 172)	−3·8% (−7·5 to 0·2)	560 (317 to 731)	22·3% (−11·8 to 59·6)
Morocco	47 (38 to 55)	42·2% (13·6 to 72·3)	783 (690 to 896)	5·1% (1·3 to 9·1)	1733 (1418 to 2051)	21·6% (−2·6 to 50·1)
Oman	5 (3 to 7)	75·1% (0·5 to 271·7)	108 (94 to 125)	7·2% (3·4 to 11·2)	203 (142 to 279)	43·3% (−10·6 to 156·2)
Palestine	4 (3 to 5)	9·5% (−19·2 to 44·7)	102 (88 to 118)	3·0% (−0·7 to 7·2)	179 (153 to 214)	−16·4% (−39·9 to 17·6)
Qatar	2 (1 to 3)	−3·9% (−37·6 to 48·3)	62 (53 to 71)	4·4% (0·1 to 8·9)	92 (67 to 122)	−10·0% (−39·0 to 32·9)
Saudi Arabia	42 (32 to 51)	88·1% (10·5 to 197·3)	854 (717 to 1 022)	7·1% (5·1 to 9·1)	1683 (1305 to 1997)	48·2% (−8·7 to 114·8)
Sudan	39 (25 to 51)	34·0% (8·6 to 64·6)	618 (540 to 719)	6·9% (2·8 to 10·8)	1882 (1092 to 2694)	21·6% (−8·3 to 64·1)
Syria	19 (15 to 23)	23·4% (−21·5 to 75·4)	413 (361 to 477)	4·0% (−0·1 to 8·3)	741 (627 to 894)	5·6% (−34·4 to 44·3)
Tunisia	19 (14 to 23)	15·6% (−22·2 to 54·7)	298 (263 to 339)	5·1% (0·8 to 8·8)	669 (530 to 820)	−3·1% (−38·2 to 27·2)
Turkey	470 (392 to 566)	−18·9% (−52·2 to 16·3)	2554 (2274 to 2864)	−1·5% (−5·7 to 2·6)	18 887 (15 336 to 23 603)	−36·1% (−64·3 to 16·7)
United Arab Emirates	26 (14 to 37)	67·0% (−4·0 to 170·1)	266 (231 to 306)	1·7% (−1·9 to 6·0)	1082 (614 to 1552)	49·8% (−12·2 to 139·8)
Yemen	21 (15 to 29)	40·1% (−0·3 to 100·8)	420 (365 to 483)	5·1% (1·0 to 9·1)	982 (665 to 1356)	20·8% (−13·6 to 68·4)
**South Asia**	**2711 (2231 to 3277)**	**48·9% (18·0 to 76·6)**	**32 104 (27 566 to 37 755)**	**14·2% (12·8 to 15·7)**	**95 154 (80 575 to 109 736)**	**35·7% (12·3 to 55·2)**
Bangladesh	146 (104 to 240)	−25·6% (−46·2 to 4·4)	2851 (2501 to 3272)	8·6% (5·0 to 12·5)	5726 (4307 to 8582)	−29·6% (−49·4 to 6·8)
Bhutan	1 (1 to 2)	30·2% (−8·1 to 75·0)	15 (14 to 18)	11·8% (7·7 to 15·7)	40 (31 to 57)	11·7% (−21·8 to 54·5)
India	2309 (1894 to 2726)	56·9% (21·4 to 86·5)	25 276 (21 450 to 30 120)	16·1% (14·6 to 17·6)	79 261 (66 554 to 90 980)	44·0% (15·8 to 65·0)
Nepal	33 (23 to 57)	45·8% (4·8 to 125·7)	514 (449 to 592)	8·1% (4·4 to 12·7)	1177 (848 to 1929)	25·4% (−9·3 to 104·3)
Pakistan	223 (175 to 311)	66·4% (18·0 to 133·3)	3448 (3020 to 3988)	7·0% (3·3 to 10·8)	8950 (7142 to 12 333)	48·5% (11·2 to 95·9)
**Southern sub-Saharan Africa**	**73 (67 to 79)**	**31·4% (6·3 to 55·4)**	**1114 (957 to 1317)**	**4·7% (3·4 to 6·2)**	**2617 (2391 to 2838)**	**23·4% (2·6 to 43·5)**
Botswana	2 (1 to 3)	42·5% (−33·1 to 133·9)	31 (27 to 36)	6·6% (3·1 to 10·1)	80 (37 to 119)	41·8% (−32·3 to 129·7)
Lesotho	2 (1 to 3)	108·8% (0·0 to 214·2)	26 (22 to 30)	6·5% (2·7 to 10·2)	63 (44 to 89)	106·2% (2·0 to 206·7)
Namibia	2 (1 to 2)	10·7% (−30·6 to 58·7)	32 (28 to 37)	4·8% (1·2 to 8·1)	63 (43 to 88)	9·0% (−29·8 to 51·3)
South Africa	55 (50 to 59)	29·3% (1·9 to 49·7)	841 (711 to 1 003)	5·9% (4·4 to 7·5)	1932 (1769 to 2108)	19·8% (−2·6 to 37·4)
Swaziland	1 (0 to 1)	30·0% (−19·4 to 101·8)	16 (14 to 19)	5·0% (1·5 to 8·8)	31 (20 to 45)	31·6% (−15·9 to 97·2)
Zimbabwe	12 (9 to 15)	38·2% (−4·2 to 153·8)	168 (145 to 197)	−1·4% (−4·7 to 1·7)	449 (356 to 574)	33·2% (−5·8 to 126·9)
**Western sub-Saharan Africa**	**273 (220 to 330)**	**16·6% (−8·0 to 36·8)**	**4075 (3541 to 4754)**	**4·5% (2·7 to 6·1)**	**13 364 (8976 to 16 996)**	**7·8% (−17·8 to 28·8)**
Benin	8 (6 to 10)	22·9% (−8·6 to 63·8)	114 (99 to 133)	3·4% (0·0 to 7·3)	336 (276 to 424)	8·3% (−22·5 to 43·6)
Burkina Faso	11 (9 to 14)	23·8% (−7·4 to 73·5)	175 (152 to 205)	5·1% (1·8 to 8·5)	545 (417 to 731)	17·2% (−11·8 to 61·9)
Cameroon	22 (15 to 28)	40·0% (−0·5 to 82·3)	235 (203 to 274)	1·5% (−1·5 to 4·5)	1004 (594 to 1407)	37·8% (−0·7 to 79·4)
Cape Verde	0 (0 to 1)	71·8% (18·9 to 124·7)	8 (7 to 9)	8·2% (4·5 to 11·9)	18 (15 to 21)	53·1% (15·3 to 95·7)
Chad	8 (6 to 11)	11·7% (−14·0 to 39·4)	140 (121 to 164)	5·2% (2·0 to 8·4)	438 (265 to 636)	10·7% (−14·9 to 39·2)
Côte d'Ivoire	23 (18 to 28)	24·2% (−7·1 to 58·9)	230 (201 to 270)	1·4% (−1·9 to 4·7)	951 (681 to 1197)	20·4% (−10·5 to 53·3)
The Gambia	1 (1 to 1)	5·1% (−16·9 to 30·9)	21 (18 to 25)	1·3% (−2·1 to 4·4)	51 (41 to 65)	−1·0% (−20·9 to 21·5)
Ghana	31 (24 to 40)	53·6% (17·4 to 147·2)	309 (269 to 357)	6·2% (2·8 to 9·8)	1186 (924 to 1531)	42·8% (8·0 to 119·1)
Guinea	9 (7 to 12)	29·8% (−12·3 to 87·6)	126 (109 to 146)	0·9% (−2·4 to 4·2)	380 (296 to 546)	11·8% (−26·7 to 62·7)
Guinea-Bissau	2 (1 to 2)	18·2% (−11·2 to 67·0)	19 (16 to 22)	2·0% (−1·4 to 5·2)	71 (54 to 97)	9·8% (−15·2 to 52·2)
Liberia	3 (2 to 3)	5·6% (−28·2 to 35·1)	43 (37 to 51)	0·4% (−2·6 to 3·8)	107 (87 to 137)	−10·1% (−42·0 to 17·3)
Mali	8 (6 to 12)	−5·7% (−34·7 to 40·1)	178 (154 to 209)	6·2% (3·1 to 9·4)	430 (327 to 594)	−13·0% (−39·8 to 33·2)
Mauritania	3 (2 to 4)	−6·1% (−32·3 to 24·6)	51 (44 to 59)	3·3% (0·0 to 6·7)	125 (80 to 166)	−3·9% (−29·6 to 27·0)
Niger	9 (7 to 14)	−5·0% (−35·3 to 51·5)	190 (165 to 223)	1·8% (−1·1 to 4·7)	450 (340 to 649)	−19·5% (−47·5 to 37·1)
Nigeria	112 (67 to 152)	4·3% (−34·5 to 38·5)	1936 (1687 to 2255)	6·0% (2·3 to 9·3)	6316 (3210 to 9266)	−1·8% (−39·0 to 32·5)
São Tomé and Príncipe	0 (0 to 0)	26·8% (−13·4 to 162·6)	2 (2 to 2)	2·8% (−0·4 to 6·2)	5 (3 to 9)	9·3% (−26·0 to 110·0)
Senegal	12 (9 to 14)	16·8% (−6·2 to 46·3)	164 (141 to 190)	2·1% (−1·3 to 5·5)	485 (398 to 592)	8·4% (−14·4 to 36·4)
Sierra Leone	5 (3 to 6)	13·6% (−12·8 to 49·5)	62 (54 to 73)	2·8% (−0·2 to 6·0)	223 (133 to 317)	−2·0% (−27·7 to 29·1)
Togo	6 (4 to 8)	19·7% (−21·1 to 60·8)	73 (63 to 85)	1·1% (−2·6 to 4·4)	245 (154 to 337)	13·2% (−26·2 to 47·8)
**Eastern sub-Saharan Africa**	**131 (104 to 214)**	**6·7% (−18·4 to 36·3)**	**3762 (3258 to 4423)**	**3·6% (2·5 to 4·8)**	**6471 (5350 to 9325)**	**−6·4% (−29·3 to 29·7)**
Burundi	4 (3 to 7)	−20·9% (−41·5 to 13·8)	98 (84 to 115)	0·7% (−2·7 to 3·8)	208 (140 to 342)	−23·5% (−43·3 to 14·4)
Comoros	0 (0 to 1)	−3·8% (−29·9 to 25·9)	9 (7 to 10)	−0·1% (−3·3 to 3·4)	15 (11 to 22)	−11·2% (−34·9 to 18·1)
Djibouti	0 (0 to 1)	23·6% (−15·4 to 87·1)	11 (10 to 13)	−0·1% (−3·2 to 2·7)	23 (16 to 33)	2·5% (−30·9 to 45·0)
Eritrea	2 (1 to 3)	17·2% (−18·3 to 66·2)	55 (47 to 64)	3·8% (0·0 to 7·6)	93 (71 to 139)	10·4% (−20·0 to 49·9)
Ethiopia	31 (22 to 61)	−8·5% (−40·0 to 54·1)	987 (857 to 1153)	3·9% (0·6 to 7·2)	1375 (1012 to 2365)	−18·5% (−48·2 to 44·2)
Kenya	15 (12 to 21)	44·15% (3·2 to 85·8)	515 (422 to 628)	3·1% (2·2 to 4·0)	731 (593 to 925)	25·0% (−2·5 to 49·1)
Madagascar	8 (5 to 14)	−4·6% (−35·0 to 30·4)	272 (236 to 316)	0·4% (−2·7 to 3·7)	405 (294 to 595)	−12·2% (−40·5 to 16·5)
Malawi	6 (4 to 11)	15·2% (−23·8 to 73·4)	164 (142 to 193)	2·1% (−1·0 to 5·4)	329 (226 to 515)	−9·8% (−41·6 to 49·5)
Mozambique	10 (6 to 19)	−0·4% (−29·7 to 32·4)	284 (246 to 333)	7·1% (3·8 to 10·7)	501 (348 to 787)	−12·3% (−39·0 to 23·6)
Rwanda	3 (2 to 8)	6·2% (−40·9 to 67·3)	110 (95 to 129)	2·9% (−0·2 to 6·3)	174 (102 to 388)	−2·2% (−41·2 to 56·6)
Somalia	3 (2 to 6)	−5·6% (−28·9 to 25·6)	93 (80 to 108)	0·1% (−3·2 to 3·4)	157 (108 to 270)	−16·1% (−39·1 to 16·3)
South Sudan	4 (2 to 7)	−1·5% (−26·8 to 31·7)	131 (114 to 153)	0·6% (−2·2 to 3·6)	198 (127 to 332)	−16·5% (−39·5 to 18·7)
Tanzania	23 (16 to 40)	11·4% (−12·3 to 43·6)	527 (455 to 615)	4·0% (0·7 to 7·4)	1170 (858 to 1765)	−3·4% (−28·3 to 28·0)
Uganda	11 (8 to 21)	17·3% (−43·2 to 112·1)	342 (294 to 407)	5·9% (2·4 to 9·3)	628 (443 to 1040)	6·8% (−48·6 to 78·1)
Zambia	10 (7 to 14)	54·2% (3·8 to 123·4)	162 (140 to 190)	3·3% (−0·1 to 7·0)	465 (318 to 666)	15·0% (−23·2 to 70·1)
**Central sub-Saharan Africa**	**40 (29 to 65)**	**−0·5% (−16·0 to 19·5)**	**1086 (943 to 1273)**	**0·8% (−1·3 to 2·9)**	**2007 (1483 to 2934)**	**−8·8% (−28·2 to 17·1)**
Angola	9 (6 to 16)	18·6% (−22·7 to 81·6)	263 (228 to 308)	5·0% (1·8 to 8·4)	497 (339 to 788)	9·3% (−24·8 to 69·3)
Central African Republic	2 (2 to 4)	5·3% (−20·1 to 36·6)	44 (38 to 52)	−0·6% (−3·9 to 2·7)	103 (67 to 159)	−0·2% (−25·6 to 32·0)
Congo (Brazzaville)	3 (2 to 4)	−9·9% (−37·5 to 20·3)	48 (41 to 55)	3·2% (−0·1 to 6·9)	115 (80 to 161)	−11·6% (−38·5 to 16·6)
Democratic Republic of the Congo	24 (16 to 43)	−4·5% (−23·2 to 14·4)	702 (606 to 823)	−1·1% (−4·2 to 1·7)	1222 (826 to 1861)	−14·5% (−36·8 to 13·5)
Equatorial Guinea	0 (0 to 1)	0·3% (−43·4 to 75·5)	9 (8 to 11)	24·6% (19·9 to 29·5)	20 (13 to 31)	−14·9% (−51·6 to 58·0)
Gabon	1 (1 to 2)	10·1% (−22·4 to 51·3)	21 (18 to 24)	2·0% (−1·5 to 5·7)	50 (37 to 66)	3·4% (−27·8 to 41·2)

95% uncertainty intervals are in parentheses. DALYs=disability-adjusted life-years. SDI=Socio-demographic Index.

**Figure 1:**
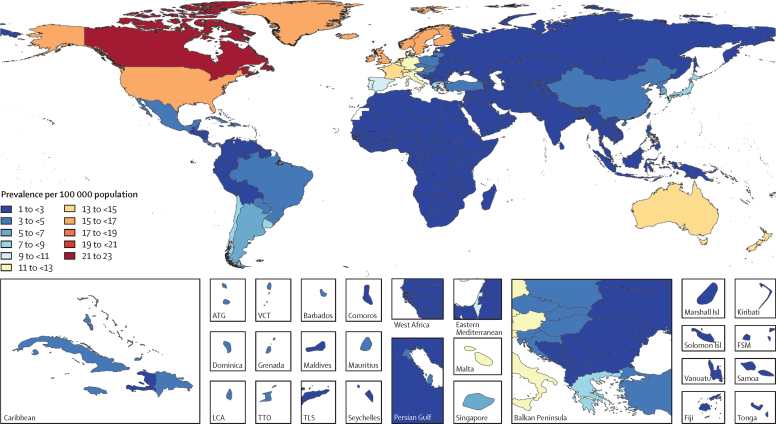
Age-standardised prevalence of motor neuron disease per 100 000 population by location for both sexes, 2016 ATG=Antigua and Barbuda. FSM=Federated States of Micronesia. Isl=Islands. LCA=Saint Lucia. TLS=Timor-Leste. TTO=Trinidad and Tobago. VCT=Saint Vincent and the Grenadines.

From 1990 to 2016, the worldwide age-standardised mortality rates of motor neuron disease increased by 8·0% (95% UI −0·1 to 12·2; table). A statistically significant increase in age-standardised mortality was present across all SDI quintiles, except for the low SDI group. Globally, the age-standardised prevalence of motor neuron disease increased by 4·5% (3·4 to 5·7; table) while the all-age prevalence increased by 18·8% (15·28 to 22·21). A map of age-standardised prevalence of motor neuron disease in each country for both sexes is shown in figure 1. Age-standardised prevalence increased in low SDI, low-middle SDI, middle SDI, high-middle SDI, and high SDI quintiles (table). The age-standardised incidence of motor neuron disease was stable in all SDI groups, except for the high SDI quintile where incidence increased by 13·0% (11·9 to 14·1) from 1·60 to 1·81 per 100 000 population (see the GBD 2016 online results tool). No statistically significant change was found in the global age-standardised DALY rate of motor neuron diseases (−1·5%, −9·3 to 2·9; table).

Considering 21 GBD regions, in 2016, the highest all-age prevalence was observed in high-income North America (20·2, 95% UI 19·0–21·5), followed by Australasia (17·7, 16·2–19·2) and western Europe (16·7, 15·3–18·1). The lowest all-age prevalence was in sub-Saharan Africa, especially in central sub-Saharan Africa (0·9, 0·8–1·1), followed by eastern sub-Saharan Africa (1·0, 0·8–1·1) and western sub-Saharan Africa (1·0, 0·9–1·2). Only a small proportion of these geographical differences in the prevalence can be explained by a different age structure of the countries: in 2016, age-standardised prevalence per 100 000 population ranged from a high of 22·6 (20·9–24·7) in Canada to a low of 1·1 (0·9–1·2) in the Central African Republic. Among regions with high SDI values, age-standardised prevalence per 100 000 population was lowest in high-income Asia Pacific, southern Latin America, eastern Europe and central Europe (figure 1). Age-standardised prevalence per 100 000 population was 16·8 (15·8–17·9) in high-income North America, 14·7 (13·5–16·1) in Australasia, 12·9 (11·7–14·1) in western Europe, 1·2 (1·1–1·4) in central sub-Saharan Africa, 1·3 (1·1–1·4) in eastern sub-Saharan Africa, and 1·3 (1·2–1·5) in western sub-Saharan Africa. Half of the world's prevalent cases resided in countries in the highest SDI quintile, most of whom were living in high-income North America and western Europe (table).

**Figure 2:**
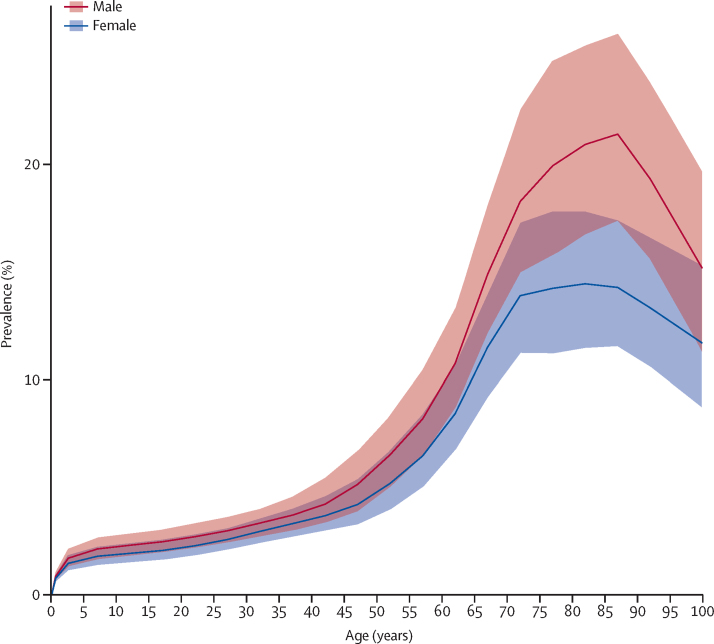
Global prevalence of motor neuron diseases by age and sex, 2016 Prevalence is expressed as the percentage of the population that is affected by the disease. Shaded areas show 95% uncertainty intervals. Values are plotted at the midpoint of 5-year age categories.

The prevalence ranking was generally consistent with that of incidence (see the GBD 2016 online results tool). The highest age-standardised incidence per 100 000 person-years was in Australasia with 2·77 (2·63 to 2·91), followed by high-income North America with 2·30 (2·20 to 2·41) and western Europe with 2·00 (1·89 to 2·11). The lowest age-standardised incidence was in western sub-Saharan Africa (0·36, 0·31 to 0·41), followed by southern sub-Saharan Africa (0·37; 0·32 to 0·43), central Asia (0·39; 0·34 to 0·44), central sub-Saharan Africa (0·39; 0·33 to 0·44), and eastern sub-Saharan Africa (0·39; 0·34 to 0·45). Prevalence was higher in males than females at all ages (figure 2), with the peak of prevalence for males at age 85–89 years, and for females at age 80–84 years. The male to female ratio of age-standardised prevalence was 1·22 (1·19 to 1·24) in 1990 and 1·25 (1·23 to 1·28) in 2016.

**Figure 3:**
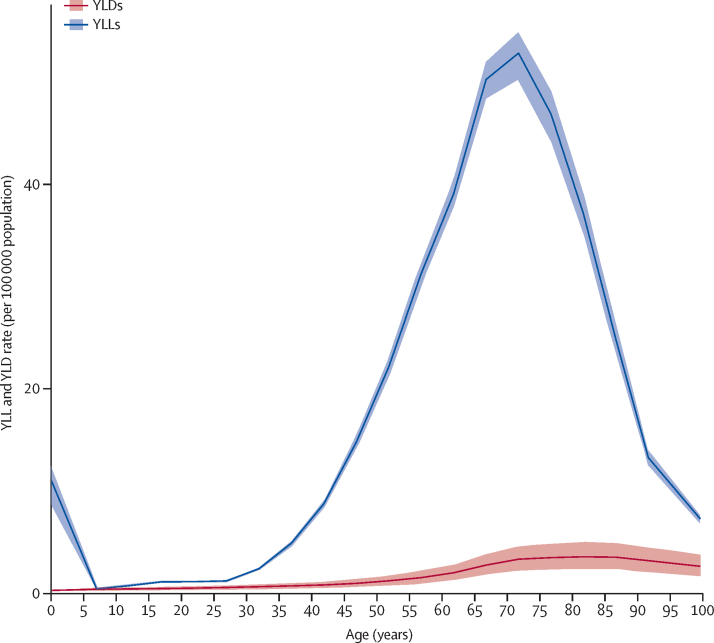
Global years lived with disability (YLDs) and years of life lost (YLLs) rates per 100 000 population due to motor neuron diseases by age, 2016 Shaded areas show 95% uncertainty intervals. Values are plotted at the midpoint of 5-year age categories.

The YLL rate curve peaked in the group aged 0–1 year and again in the group aged 70–74 years (figure 3). YLD rates showed a steady increase to a plateau at age 80–84 years (figure 3). The YLD rates were much lower than the YLL rates, reflecting the high case fatality of motor neuron disease.

**Figure 4:**
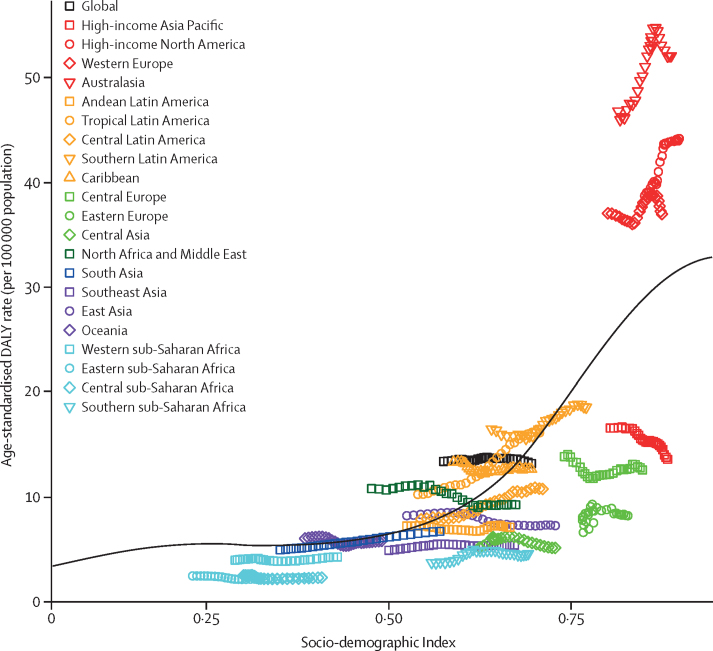
Age-standardised disability-adjusted-life-year (DALY) rates for motor neuron diseases by 21 Global Burden of Disease regions by Socio-demographic Index (SDI), 1990–2016 The black line represents expected values based on SDI from a regression of all location data over the entire 1990 to 2016 estimation period.

The expected age-standardised DALY rates increased with SDI (figure 4), from about five per 100 000 population in countries with a very low SDI to about 30 per 100 000 population in countries with a high SDI. The change in DALYs as SDI increased was greatest at high SDIs. The rates in high-income North America, western Europe, and Australasia were higher than expected based on their SDI levels. By contrast, the age-standardised DALY rates in the high-income Asia Pacific region were lower than expected based on its SDI. None of the 84 risk factors analysed in GBD 2016 were shown to have sufficient evidence of association with motor neuron disease incidence or deaths.

## Discussion

More than half of global deaths and close to half of all prevalent cases of motor neuron diseases occurred in three high-income regions: North America, western Europe, and Australasia. Motor neuron disease cases and deaths were far fewer in other parts of the world. Globally, from 1990 to 2016, the all-age prevalence increased more than the age-standardised prevalence, indicating that the largest part of the global prevalence increase was due to ageing.

The geographical differences in age-standardised incidences might be partly due to more accurate case ascertainment and diagnosis of motor neuron disease in high SDI countries compared with countries with low and middle SDIs. For example, a significant geographical difference in motor neuron disease prevalence was observed in the USA and western Europe between regions with different levels of quality of access to health-care systems.^10^ However, high SDI countries in the Asia Pacific region, where an accurate diagnosis of motor neuron disease is more probable than in low SDI countries, had a lower incidence than high SDI countries in western Europe and North America. This geographical heterogeneity suggests that the differences in the prevalence and incidence of motor neuron disease might be due to ethnicities and ancestries, and that the apparent relationship with SDI might be spurious. In a 2017 study in which subcontinents were considered as surrogates of ancestries, a higher prevalence of amyotrophic lateral sclerosis was reported in Europe, the USA, and New Zealand than in east Asia.^8^

The increase in age-standardised incidence of motor neuron diseases in the high SDI regions during the study period might, in part, be due to improved diagnosis, whereas the increase in age-standardised prevalence is probably due to improved survival. In particular, non-invasive ventilation can prolong survival^21^ and has been increasingly included in the usual practice of multidisciplinary motor neuron disease centres. The improvement of quality and reduction of time to certainty of amyotrophic lateral sclerosis diagnosis might also appear to increase the duration of disease.^22^ Additional contributing factors could include an increase in the public awareness of amyotrophic lateral sclerosis (including that generated by major clinical trials launched since the early 1990s in Europe and the USA), and increased awareness that progressive weakness is not a normal part of ageing.

We found that the prevalence of motor neuron disease increases particularly after age 50 years, with a peak at around age 85 years, followed by a rapid decline in males, while in females the curve is flat between age 70 and 85 years, followed by a decline (figure 2). The rapid decline in the oldest age group (ie, ≥85 years) could be due to poor ascertainment because of more complex clinical features or comorbidities, competing mortality from other causes (eg, cardiovascular disease, dementia), or both. Older people are less frequently referred to tertiary neurological care^11^ and are less likely to get a correct diagnosis than are younger patients. Another factor that might play a part in the observed decline in the prevalence of motor neuron disease in the older population relates to different clinical expression of the disease, with a greater case fatality in this age group (eg, more frequent bulbar motor neuron disease cases).^11^, ^23^

Our findings suggest that the prevalence of motor neuron disease is consistently higher in males than females across all age groups, with no changes in the male to female ratio between 1990 and 2016. The ratio is similar to that in studies from European registries, although in those studies incidence of amyotrophic lateral sclerosis in females increased over time.^10^ The reported change in sex ratio of amyotrophic lateral sclerosis in the earlier study^11^ has been attributed to changes of exposure for females to possible environmental factors, such as smoking. In GBD, however, the evidence for an association of smoking with all motor neuron disease was considered insufficient.^21^

Previously reported prevalence per 100 000 population for amyotrophic lateral sclerosis (5·40 in Europe, 3·40 in the USA, and 2·34 in Asia)^7^ are lower than the GBD 2016 estimates of overall motor neuron disease prevalence per 100 000 population (10·00 in Europe, 19·37 in the USA, and 3·13 in Asia), as expected, because we included additional motor neuron diseases.

This study explored motor neuron diseases in all age groups. About 14% of cases were younger than age 20 years, an approximate estimate of the genetic forms with early clinical onset. The inclusion of spinal muscular atrophy, including all its clinical and genetic phenotypes, has had an effect on DALYs, with the presence of a peak in the first year of life in addition to the second peak in people aged 70 years and older due to amyotrophic lateral sclerosis. However, the inclusion of spinal muscular atrophy and other motor neuron diseases of early life has not produced a substantial change in the age prevalence curve compared with studies including only amyotrophic lateral sclerosis.

Incidence and prevalence of motor neuron disease in early childhood (ie, spinal muscular atrophy and hereditary spastic paraplegia) seems to be lower in GBD 2016 estimates than previous estimates. For example, a prevalence of approximately one to two per 100 000 population and incidence of one in 10 000 livebirths per year have been estimated with spinal muscular atrophy type I (60% of cases).^24^ A previous report in England, Finland, Norway, and Hungary estimated a birth incidence of spinal muscular atrophy of ten per 100 000 livebirths.^25^ A larger survey,^17^ done in 2015, which took data from 27 population-based studies from Europe, Middle East, North America, Asia, South America, Africa, and Australia, reported a similar range, from 5·1 to 16·6 cases per 100 000 livebirths. We found an incidence of 1·9 per 100 000 person-years in the group aged 28 days to 1 year, indicating a relevant risk and burden in the first year of life (see the GBD 2016 online results tool). Age-specific incidence in this age group varied notably among locations: from 0·9 (sub-Saharan Africa) to 13·1 (Australia) per 100 000 person-years. The prevalence of spinal muscular atrophy is established, as for other genetic diseases of early years of life, by several factors, including family size of the proband, the availability of genetic counselling, prenatal diagnosis with genetic testing, and access to and willingness to have pregnancy termination. Additionally, spinal muscular atrophy frequency seems higher in white populations compared with other ethnic groups, similar to amyotrophic lateral sclerosis.^26^, ^27^

Several limitations of the study should be considered. First, given the diagnostic challenges of motor neuron diseases, some categories of motor neuron disease are probably still underdiagnosed, especially in older people and ethnic minority groups.^28^ Although the criteria for amyotrophic lateral sclerosis diagnosis in adults changed several times over the study period,^29^ we did not detect a systematic bias between data based on El Escorial criteria and data based on alternative case definitions. Second, data are scarce from large parts of the world, including sub-Saharan Africa, most of Latin America, eastern Europe, and south and central Asia. Despite the fact that the Asian population constitutes more than 50% of the world population, few epidemiological studies of motor neuron diseases have been done outside Europe and North America.^30^ The data indicate a difference in the prevalence and incidence of motor neuron disease, with lower estimates in Asia, Latin America, and Africa, as compared with the rest of the world. This difference might, at least in part, reflect missed diagnosis because of the absence of appropriate diagnostic instruments. Third, mortality rate estimations were based on data classified using ICD-9 and ICD-10. Although both versions of the ICD are highly congruent in the field of motor neuron disease, we cannot exclude an influence of the classification evolution, even if unlikely. As prevalence is related to incidence and disease duration, we should consider that variation in prognosis between geographical area, especially due to variation in care, might explain part of the prevalence variation. For example, tracheostomy is done in about 30% of Japanese patients, whereas in the USA and Europe proportions range between 0% and 10%.^31^, ^32^ Conversely, use of non-invasive ventilation appears much higher in the USA (15–35% of cases) as compared with Japan and Europe.^33^

This report is one of the most accurate so far on motor neuron disease epidemiology, but is still based on inference for large areas. Therefore, further improvements in estimates of the burden of motor neuron disease will require new research in these areas of the world; prevalence and possibly incidence studies in low-middle-income and low-income countries, particularly in sub-Saharan Africa, Latin America, the Caribbean, and Asia, are needed. Epidemiological studies of subpopulations of countries with diverse ethnicities are also important, given the supposed lower motor neuron disease incidence in mixed populations as compared with homogeneous populations.^34^

Our estimate of the severity of disability is based on the PRO-ACT database and therefore indicative of disability in amyotrophic lateral sclerosis and not in spinal muscular atrophy and other motor neuron diseases. Another limitation is that participants in randomised trials are characterised by a less severe form of amyotrophic lateral sclerosis than are participants from population-based studies.^35^ Finally, no data exist on the effect of cognitive impairment on disability, an important clinical trait of amyotrophic lateral sclerosis that affects about 50% of individuals at diagnosis,^36^ and this might have led to underestimation of the overall motor neuron disease disability.

Unfortunately, no effective treatment for motor neuron diseases exists. The only licensed drugs for amyotrophic lateral sclerosis are riluzole and, more recently, edaravone. Even if these drugs change the survivorship, the effect is limited to a few more months of life, and the effect on the disability caused by amyotrophic lateral sclerosis is not clear.^37^, ^38^, ^39^ Conversely, a promising new approach exists for gene-modifying therapy for spinal muscular atrophy.^40^

These data show that, with ageing of the world population, the burden of motor neuron diseases on health services is likely to increase substantially in coming decades. New epidemiological studies in areas without data are needed. Epidemiological studies in diverse populations might improve estimates of motor neuron disease frequency, understanding of disease phenotypes and risk factors, and the completeness of case ascertainment and referral.^41^ In the meantime, with limited possibilities for effective medical intervention, these GBD 2016 data are of relevance to health-care planning and resource allocation for the extensive care needs of people with motor neuron diseases.
